# Expanding the toolkit: OPMs for studying the developing brain

**DOI:** 10.1162/IMAG.a.1387

**Published:** 2026-10-01

**Authors:** Margot J. Taylor, Natalie Rhodes

**Affiliations:** Diagnostic & Interventional Radiology, Hospital for Sick Children, Toronto, Canada; Program in Neurosciences & Mental Health, Research Institute, Hospital for Sick Children, Toronto, Canada; Department of Medical Imaging, University of Toronto, Toronto, Canada; Department of Psychology, University of Toronto, Toronto, Canada

**Keywords:** optically pumped magnetometers, magnetoencephalography, neurodevelopment, toddlers, infants

## Abstract

Tremendous maturation in brain structure and function, underlying very rapid cognitive development, occurs in the early years of life, yet it has been difficult to obtain reliable measures of brain function over this period. A major challenge has been the difficulty of acquiring reliable neuroimaging data from awake young children, who often struggle to tolerate conventional imaging environments or remain still during scanning. Existing approaches each involves important compromises between spatial and temporal resolution, movement tolerance and practicality for use in early childhood. Optically pumped magnetometers (OPMs), a recent advance in magnetoencephalography (MEG) technology, are expanding the developmental neuroimaging toolkit. By enabling adaptable, wearable, and movement-tolerant MEG systems, OPMs retain the excellent spatial and temporal resolution of conventional cryogenic MEG technology while improving suitability for infants, toddlers, and young children. In this commentary, we briefly review the current functional neuroimaging toolkit used across development and discuss how OPMs are transforming the study of the developing brain. We highlight recent applications of OPMs to study early life, their potential for investigating brain–behaviour relationships and social interaction, and their promise for improving inclusion of clinical populations traditionally underrepresented in neuroimaging research. We also outline remaining technical and methodological challenges that should be addressed as the field continues to grow.

## 1 Introduction

Early childhood is one of the most important yet least understood periods of human brain development. Much of what makes us human develops rapidly over our first few years of life: we learn to walk, talk, and interact with one another. The lack of understanding of brain function in this period of life can largely be attributed to limitations in existing neuroimaging tools and the challenge in using these techniques in young children who may still be preverbal, cannot yet follow instructions (or in the “terrible-twos”, choose not to), and generally have difficulty staying still and motionless for any period of time while awake. Sleep studies have been the recourse for MRI and fMRI studies, but that seriously limits assessment of cognitive function, and even sleep studies are best done in those under 1 year of age. Thus, to date, although we have extensive behavioural studies on the emerging skills in the preschoolers (typically those 5 years and under), we have precious little knowledge on the underpinning brain function, compared with the vast quantity of studies on school-aged children, adolescents, and adults. Below we will briefly review available neuroimaging options that have been tried with the preschool age range and then present optically pumped magnetometers (OPMs), highlighting their value in facilitating functional neuroimaging, especially in toddlers and young children.

## 2 The Current Toolkit for Functional Neuroimaging Across Development

### 2.1 Functional MRI

Functional MRI is the workhorse for functional brain imaging in adults, due to its excellent spatial resolution and availability in many research and medical centres (particularly in the global west). fMRI has also provided a wealth of knowledge about the development of functional brain networks from foetal and infant studies to later childhood. As fMRI is highly sensitive to motion and is a loud and intimidating scanning environment, this can be prohibitive for toddlers, limiting study largely to sleep protocols. For this reason, the majority of fMRI developmental studies are from early in infancy, when the infants/toddlers are tested asleep, or in later childhood (Azhari et al., 2020; Hendrix & Thomason, 2022). For clinical paediatric populations as well, especially those with sensory difficulties (e.g., autism), fMRI is challenging.

### 2.2 fNIRS

Functional near infrared spectroscopy (fNIRS) offers a more practical alternative for a young age range as it offers a wearable helmet and allows some movement during testing. As with fMRI, fNIRS measures blood flow as a proxy of brain activity. Due to ease of use, however, there are many studies across early childhood using neural metrics derived from fNIRS. Nevertheless, it is a coarse measure of brain activity as it suffers from poor spatial and temporal resolution and is only sensitive to superficial layers of the cortex (see Hoshi, 2016; Scarapicchia et al., 2017, for reviews). fNIRS is also impacted by hair colour and thickness, as well as melanin content in the skin (Kwasa et al., 2023), limiting its generalisability across ethnic groups.

### 2.3 EEG

EEG is arguably the most widely used functional neuroimaging tool across early life, largely due to its accessibility; EEG systems are relatively inexpensive, portable, and tolerant of some movement. Thus, EEG has provided decades of insights into neurodevelopment. However, signals are distorted due to the skull and scalp, which limits the spatial resolution; consequently, although it provides excellent temporal resolution, accurately determining the sources of the recorded activity is difficult. EEG is also sensitive to muscle artefact (Muthukumaraswamy, 2013), which interferes with recording gamma band activity (Boto et al., 2019).

### 2.4 Cryogenic magnetoencephalography (MEG)

MEG also benefits from excellent millisecond temporal resolution and benefits from higher spatial resolution than EEG, due to minimal distortion of magnetic fields from the skull and scalp (Baillet, 2017) and lower susceptibility to muscle artefact (Muthukumaraswamy, 2013), making it the ideal modality for real-time functional brain imaging. Although there have been many developmental studies in awake children with cryogenic MEG, these, with few exceptions (e.g., Chen et al., 2022; Clarke et al., 2022; Otten et al., 2025), have mostly been limited to school-aged children. This is due to the cryogenic MEG systems relying on superconducting quantum interference devices (SQUIDs). These sensors must be held in a bath of liquid helium, requiring a rigid one-size-fits-all helmet, typically sized for a large adult head. Thus, scanning young children results in a large sensor-to-brain distance, reducing signal-to-noise ratios. Further, MEG is very sensitive to movement while in the helmet, with small movements (millimetres) reducing spatial fidelity (which can be compensated post-hoc using SSS) and larger movements (centimetres) prohibiting source reconstruction. As with fMRI, this further limits its use with infants, toddlers, and young children who can often not sit or lie still long enough to complete the tasks. Although there are child-sized cryogenic MEGs (Okada et al., 2016; Roberts et al., 2014), there remain very few globally and they still have the constraints of the child remaining almost perfectly still during scanning.

## 3 Next Generation MEG

With the recent advent of optically pumped magnetometers (OPMs), all the benefits of MEG—the excellent frequency information, temporal and spatial resolution—are maintained, but OPMs are not cryogenic and thus do not need to be within a fixed helmet, but can be adapted to different head sizes, can be made wearable, and consequently enable movement. OPMs manipulate the quantum properties of alkali metals or helium gas to measure tiny changes in magnetic fields. There are broadly two types of commercial OPMs for MEG, Rubidium-87 based and Helium-4 based. For a review on the principles behind the Rubidium OPMs, see Tierney et al. (2019). For a description of the current generation of Helium OPMs, see Bonnet et al. (2025).

Following the first demonstration of OPM recordings in humans 20 years ago (Xia et al., 2006), the development of OPM-MEG has been remarkably rapid in recent years. Sensors were miniaturised (Boto et al., 2017; Osborne et al., 2018), incorporated into multi-channel wearable systems (Boto et al., 2018) and subsequently into whole-head neuroimaging tools (Alem et al., 2023; Bonnet et al., 2025; Hill et al., 2020) in a few short years.

Without the requirement for insulation from cryogens, the sensor-to-brain distance is reduced with OPM compared with cryogenic MEG, and thus the magnetic field strength and signal-to-noise ratio are improved, especially with small heads. This in turn leads to improved sensitivity to deep brain structures.

Central to the utility of OPM-MEG is the ability to wear, and, therefore, move, with the sensors, which provides the additional challenge of sensitively controlling the magnetic field environment. Magnetometers that move through an ambient magnetic field will measure the change in magnetic field, which can introduce artefacts or saturate sensors that have a low dynamic range. This has been addressed through a combination of multi-layer magnetically shielded rooms, often beyond the typical cryogenic MEG needs, and activate magnetic field nulling coils, which dynamically compensate for the environmental magnetic fields (Holmes et al., 2019). These advances mean that OPM systems can tolerate large head movements that would typically render cryogenic MEG data unusable, a critical step towards practical neuroimaging in young children. Also, although metallic dental work is less common in young children, OPMs are much more resilient to magnetised materials in the participants’ head (mouth, typically) (Safar et al., 2024), as the head moves with the sensors. In contrast, such implants cause significant artefact in cryogenic MEG.

It is this flexibility that first enabled the demonstration of OPMs as lifespan compliant (Hill et al., 2019); by simply moving OPM sensors between different sizes of adapted bicycle helmets, the same sensors could measure neural signals from a 2-year-old, 5-year-old, and adult. The 7 years since this first demonstration have seen substantial and diverse developments and applications of OPMs in developmental neuroscience: from paediatric epilepsy (Feys et al., 2023, 2022) to beta and gamma oscillations (Rhodes et al., 2025; Rier et al., 2024), aperiodic activity (Vandewouw et al., 2024), emotional face responses (Safar et al., 2025), and auditory evoked activity (Corvilain et al., 2025).

Due to the novelty of this technology, the systems used vary substantially, with examples shown in Figure 1. Paediatric OPM systems include rigid systems that resemble cryogenic MEG, flexible EEG-like caps, semi-rigid arrays of sensors, and fixed helmets with moveable sensor holders to wearable bicycle-style helmets. Imaging across development with a single system is a particularly challenging engineering problem: while a 1-month-old infant cannot support its own head, necessitating supine scanning configurations, a 2-year-old may struggle to remain still even briefly. No single system has yet resolved these constraints simultaneously, and the choice of OPM platform currently involves trade-offs between sensor coverage, proximity to the scalp, weight, thermal management, and age appropriateness or wearability.

**Fig. 1.**
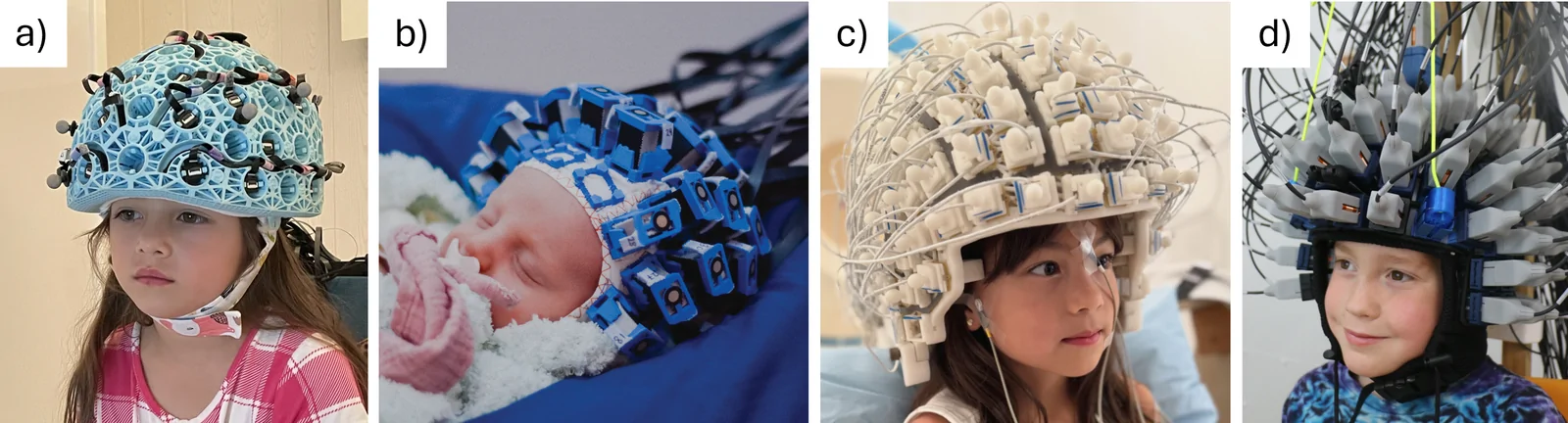
OPM systems for studying children. (a) A 5-year-old child in a 3D-printed size-matched helmet with a 128-channel Cerca Magnetics system (The Hospital for Sick Children, Toronto, Canada). (b) A flexible EEG-like cap containing OPMs (QuSpin Inc.) worn by an 1-month-old infant (Corvilain et al., 2025). (c) A child wearing the 128-channel FieldLine HEDscan system (Edgar et al., 2026). (d) An 8-year-old child wearing the 96-sensor Mag4Health system (Aston University, Birmingham, UK).

## 4 The Potential of OPMS in Developmental Neuroscience

### 4.1 Imaging the moving child

Neuroimaging in early life has routinely been hindered by lack of motion tolerance, meaning experimental design must restrict naturalistic motion that is intrinsic to how children interact with and learn from their environment. With appropriate magnetic field control, it is feasible with OPMs to implement interactive, naturalistic paradigms that would be impossible with conventional neuroimaging approaches. OPMs are also substantially less sensitive to muscle artefact than EEG, meaning that even when a child is moving, there is less signal degradation (Boto et al., 2019; Seedat et al., 2024). This dual advantage of both movement and muscle activity tolerance opens doors to paradigms previously confined to fNIRS or behavioural-only assessment.

OPMs could substantially advance the study of complex motor tasks across development (Holper et al., 2009; Teng et al., 2025). Motor paradigms have been demonstrated with OPMs in adults (e.g., Hill et al., 2019; O’Neill et al., 2025; Rea et al., 2022; Spedden et al., 2025), and their extension to children would allow investigation of motor learning, neural correlates of performance in studies of motor function and rehabilitation (Hao et al., 2024), and joint activities (Su et al., 2022), domains most often investigated with fNIRS; OPMs could provide richer spectral and spatial information. For example, the play-based assessments of the Mullen Scales of Early Learning (Mullen, 1995) could be conducted while the child is wearing an OPM helmet or cap, increasing our understanding of the brain networks underlying a young child learning to draw or count. A first move towards this would be to use tasks from the Mullen, such as object manipulation or joint attention tasks, to establish the feasibility of this approach.

Beyond complex paradigms, even basic developmental neurophysiology across the early years of life remains incompletely characterised. Changes in the range of periodic signals (peak frequency shifts, spatial localisation changes with age, etc.), the aperiodic signal, and the range of time-locked evoked or induced responses have not yet been systematically mapped across the preschool years with the spatial and spectral resolution that OPMs can provide. These normative neurophysiological metrics, which undergo substantial maturational changes in typical development, may serve as early markers for an atypical developmental trajectory. Establishing such a normative foundation is a critical prerequisite for studies that include clinical populations, where early identification of a neurodevelopmental condition allows earlier intervention, which is a huge benefit to improving the quality of life of the child and the family (Brian et al., 2015; Guthrie et al., 2023). Finally, as OPMs are lifespan compliant, they allow longitudinal studies, as well as cross-sectional studies, across wide age ranges, using identical neuroimaging systems, providing reliable and consistent data to better understand subtleties in neurodevelopment.

### 4.2 Social neuroscience and hyperscanning

Social interactions are fundamental to human society, and the acquisition of social behaviours across early childhood is essential for a child’s long-term well-being. Face processing and the understanding of facial emotion are well-researched areas, yet few neuroimaging studies have successful linked neural responses with active social behaviour in very young children. The increased interactivity afforded by OPMs will enable richer paradigms such as live face-to-face interactions or dynamic social stimuli, deepening our knowledge of how children learn to process faces and their expressions in real time.

An additional exciting frontier made possible by OPMs is the ability to simultaneously record from multiple interacting participants, or *hyperscanning*. This is particularly important for improving our understanding of social developmental neuroscience, where the neural dynamics of dyadic interactions, such as those between an infant or young child and a parent, or between two children at play, remain largely unexplored at the timescales MEG can resolve (although cryogenic MEG hyperscanning has been explored using video or mirror systems, see Hirata et al., 2014). Adult OPM-MEG hyperscanning has already been demonstrated (Holmes et al., 2023), providing the technical proof-of-concept. Extending this to child–parent or child–child dyads would allow researchers to study the brain-to-brain synchrony that underlies shared attention, turn-taking, joint reading, and language acquisition in more naturalistic settings. A parent reading a picture book to a toddler, for example, offers an ecologically valid window into the neural mechanisms of early literacy and shared narrative understanding that conventional neuroimaging techniques could not fully capture.

### 4.3 Clinical populations

Neuroimaging has historically had to exclude those deemed challenging to scan due to inability to tolerate the MRI environment, the noise and confinement it entails, or the application of EEG electrodes. This has certainly resulted in biases in clinical samples to those who show less severe phenotypes, limiting the generalisability of findings to the full spectrum of a given condition. There has been an increasing push to try to include lower-functioning children in neuroimaging studies (e.g., Kuschner et al., 2021), and OPMs are well placed to facilitate this. The increased tolerability to movement, the relative ease of placing and removing OPM helmets or caps compared with EEG net preparation or MRI setup, and the absence of acoustic noise make MEG with OPMs a more accessible environment for children with sensory sensitivities or behavioural challenges.

## 5 Remaining Challenges

Despite their promise, OPMs present several technical and methodological challenges that should be addressed before they fulfil their full potential as a developmental neuroimaging tool. It is also necessary to note that although OPM systems are far less expensive than cryogenic MEG systems, they are still more expensive than EEG, and this likely limits their use to specialised research centres in the near future.

### 5.1 Array design

The systems currently available either prioritise wearability at the cost of consistent on-scalp sensor placement and requirement to move sensor arrays between different sized helmets, or they prioritise on-scalp placement at the cost of lightweight, wearable design. An ideal array design for lifespan neuroimaging with OPMs has not yet been established. An ideal OPM-MEG system for scanning young children requires sufficient sampling across the head to prevent spatial sampling gaps (Boto et al., 2022), appropriate heat management for rubidium-based sensors (which operate at elevated temperatures), acceptable overall weight for all OPM types, practical cable management, and a child-friendly exterior to reduce anxiety and encourage compliance. Achieving all of these simultaneously within a single system remains an open engineering challenge.

### 5.2 Standardisation across systems and sites

Developmental neuroimaging has benefitted enormously from large, open-access, multi-site datasets such as the Adolescent Brain and Cognitive Development (ABCD) study (Casey et al., 2018), which have accelerated discovery by providing statistical power and sample diversity that no single site could achieve alone. Currently, there are no community-standardised OPM-MEG acquisition protocols or end-to-end analysis pipelines that would enable equivalent cross-site integration on this scale. Given the rapid increase in OPM uptake and the high probability that these systems will replace cryogenic MEG systems in many centres over the coming decade, establishing common methods, shared data formats, and harmonised pre-processing pipelines should be a priority for the community.

### 5.3 Integration with complementary measures

Many research questions in developmental neuroscience require simultaneous acquisition of complementary physiological and behavioural measures. Eye tracking, for example, enables the tracking of attention and gaze behaviour in pre-verbal children who cannot provide a verbal or cued response (e.g., a button press); its integration with OPM-MEG would allow robust linking of neural activity to visual gaze behaviour and pupillometry in naturalistic paradigms (Hepach & Westermann, 2016). While OPM-compatible eye trackers are available on the market, further work is needed to integrate the advances in rapid calibration and infant/child-friendly acquisition with the magnetic compatibility required for magnetometers. Similarly, integration with cardiac (MCG) and muscular (MMG) metrics would provide meaningful indices of autonomic state and allow for muscle activity to be tracked in movement paradigms, as demonstrated in Spedden et al. (2025).

An additional advantage of wearable OPM systems is their compatibility with external motion capture and video-based behavioural analysis tools. Motion tracking using systems such as OptiTrack has already been incorporated into OPM experiments to continuously monitor participant movement and sensor positioning (Roberts et al., 2019; Seymour et al., 2021). Likewise, markerless video-analysis approaches based on tools such as OpenPose offer the potential to quantify body posture (O’Neill et al., 2025) and thus social interaction dynamics alongside neural recordings. These developments are particularly important for developmental neuroscience, where behaviours such as play are inherently dynamic and difficult using conventional trial-repeat techniques. The combination of OPM-MEG with behavioural tracking technologies, therefore, offers a powerful framework for investigating brain activity during real-world social and motor behaviour in infants and young children. The development of compatible protocols and synchronisation for these supplementary measures alongside OPM-MEG recordings would be an important next step for the field.

## 6 Conclusions

OPM technology has the ability and potential to transform neuroimaging research in toddlers, young children, and developmental clinical populations. The customisable helmets or caps are easier to put on (and take off), they can accommodate natural movement that is essential for engaging young children in meaningful tasks, and they retain the superb temporal and spatial resolution of cryogenic MEG, without the practical and financial burden of liquid helium infrastructure. OPMs are already advancing our understanding of brain–behaviour relations over the early years of life, and we expect a surge in data in the near future. The field is still in its infancy, but the progress is rapid. With OPMs added to our neuroimaging toolkit, the window into the earliest years of brain development is, at last, beginning to open wide.

## Data Availability

No new data or code was generated or analysed in support of this article.
